# The Cure Rate after Placebo or No Therapy in American Cutaneous Leishmaniasis: A Systematic Review and Meta-Analysis

**DOI:** 10.1371/journal.pone.0149697

**Published:** 2016-02-19

**Authors:** Gláucia Fernandes Cota, Marcos Roberto de Sousa, Tatiani Oliveira Fereguetti, Priscila Said Saleme, Thais Kawagoe Alvarisa, Ana Rabello

**Affiliations:** 1 Clinical Research and Public Policy in Infectious and Parasitic Diseases, Centro de Pesquisas René Rachou, Fundação Oswaldo Cruz, Fiocruz, Belo Horizonte, Minas Gerais, Brazil; 2 Post-Graduate Program in Adult Health Sciences, Universidade Federal de Minas Gerais, Belo Horizonte, Minas Gerais, Brazil; 3 Hospital Eduardo de Menezes, Fundação Hospitalar do Estado de Minas Gerais-FHEMIG, Belo Horizonte, Minas Gerais, Brazil; The George Washington University School of Medicine and Health Sciences, UNITED STATES

## Abstract

**Introduction:**

There are few drugs with proven efficacy in cutaneous leishmaniasis (CL), and pentavalent antimonial derivatives are still the main first-line therapeutic agents worldwide, despite their recognized high toxicities. Randomized controlled clinical trials assessing the efficacy and safety of new therapeutic modalities are of high priority, and the definition of the design of such trials raises debate about the use of placebo as a comparator. To support the use of placebo as a comparator, two main points need to be addressed: 1- the cure rate without any therapeutic intervention and 2- the damage caused by CL and its impact on patients.

**Objective:**

The aim of this study was to systematically assess the spontaneous cure rate for American CL and to broaden the discussion about placebo use in CL trials.

**Methods:**

The PRISMA guidelines for systematic reviews and the Cochrane manual were followed. The sources used were the PubMed and LILACS databases. Studies were included if they reported cure rates using placebo or no treatment in American CL.

**Results:**

Thirteen studies of a total of 352 patients were ultimately included in this review. The summarized global cure rates for all *Leishmania* species according to the intention-to-treat analyses performed at approximately three (“initial cure”) and nine (“definitive cure”) months after “no treatment” or placebo use were 26% (CI95%: 16 to 40%) and 26% (CI95%:16 to 38%), respectively. Notably, a significantly lower cure rate was observed for *L*. *braziliensis* infection (6.4%, CI95%:0.2 to 20%) than for *L*. *mexicana* infection (44%, CI95%:19 to 72%), p = 0.002. Of note, relapse occurred in 20% of patients with initial healing (CI95%:9.2 to 38.9%).

**Conclusion:**

These results clearly demonstrate a low spontaneous cure rate following no-treatment or placebo use, confirming that this strategy for the control group in CL studies expose patients to greater morbidity, especially for CL caused by *L*. *braziliensis*. Therefore, from this point, the crucial questionto consider regarding placebo use isthe seriousness of the suffering caused by this disease.

## Introduction

Cutaneous leishmaniasis (CL) is considered one of the most neglected and serious parasitic infectious skin diseases in many developing countries. Infectious diseases can be considered neglected “when there is a lack of effective, affordable, or easy to use drug treatments” [1].

The current recommendations for CL treatment are generally weak, as the quality of the supporting evidence for such treatments is generally inadequate [2, 3]. Although three major systematic reviews [4–6] have shown that treatment with antimonial derivatives was superior to the other options available, their low cure rate (76.5%) [6] and the recognized toxicity of antimonial derivatives clearly indicate the urgent need for better treatment alternatives. Moreover, high-quality clinical trials testing new treatment modalities with reliable evidence of efficacy, safety, tolerability and effectiveness are absolutely necessary.

In addition to being scientifically valid, clinical trials must be ethical and minimize the risks to which the research participants are exposed. In the case of CL, to understand the risks that participants of studies that compare different interventions are exposed to, the spontaneous cure rates for CL caused by each species of *Leishmania* in different endemic regions must be determined. Based on a non-systematic literature observation, the spontaneous healing trend reported in the Old World—especially with *L*. *major* infection[7]- is far lower than that observed among American CL patients (for CL caused by organisms of the *L*. *mexicana* complex and Viannia sub-genus–the *L*. *braziliensis* and *L*. *guyanensis* complexes). This suggests that the frequency of spontaneous healing observed for one species should not be generalized to other species. The aim of this study was to systematically assess the rate of spontaneous cure in American CL to support the planning of future clinical studies with regard to statistical parameters and placebo use in the Americas.

## Data sources and study selection

The review methodology followed the recommendations of the PRISMA guidelines [8] for systematic reviews (S1 Text) and the recommendations of the Cochrane Collaboration Group [9]. We searched PubMed and Lilacs, the free access bases, with no language restriction from inception through March, 2015 to identify all studies that described the rate of spontaneous cure in American CL. Studies were included if they reported CL cure rates with placebo or no treatment in individuals living in the Americas. The search was planned independently by two reviewers (GFC and MRS) who proposed three different strategies for PubMed database: (i) a comprehensive strategy, (ii) a strategy directed at the “placebo” term and (iii) a strategy using all narrative and systematic reviews on the subject as the basis. Study selection was made independently by four reviewers (GFC, TFO, PSS, and TKA), and any disagreement was resolved by consensus. In the first strategy, we used a combination of the following keywords: "leishmaniasis, cutaneous" [MeSH Terms] OR ("leishmaniasis"[All Fields] AND "cutaneous" [All Fields]) OR "cutaneous leishmaniasis" [All Fields] OR ("leishmaniasis" [All Fields] AND "cutaneous"[All Fields]) OR "leishmaniasis, cutaneous" [All Fields] AND ("drug therapy" [Subheading] OR "drug" [All Fields] AND "therapy" [All Fields]) OR "drug therapy"[All Fields] OR "drug therapy" [MeSH Terms] OR ("drug"[All Fields] AND "therapy"[All Fields]) AND "humans" [MeSH Terms]. In the second strategy, we used a combination of the following keywords: "leishmaniasis, cutaneous" [MeSH Terms] OR ("leishmaniasis"[All Fields] AND "cutaneous"[All Fields]) OR "cutaneous leishmaniasis"[All Fields] OR ("leishmaniasis"[All Fields] AND "cutaneous"[All Fields]) OR "leishmaniasis, cutaneous"[All Fields] AND "placebo"[All Fields]). Finally, in the third strategy, studies with some arm of “no treatment” or placebo were searched for in the reference lists of all reviews identified by the first two strategies. The initial search was complemented by a manual search of the reference lists from the retrieved articles. Studies were considered eligible if they were presented in an original article and examined American patients with CL diagnosis treated with placebo or left untreated. There were no restrictions on the publication language, date of publication or study design. Studies involving non-human participants and studies with less than ten patients in the non-treatment arm were excluded. Studies using any substance with intrinsic activity in placebo arm were excluded. The full texts of the selected articles were read to confirm their eligibility and to extract the data. Four investigators (GFC, TFO, PSS, and TKA) independently extracted the participant data, studies characteristics, predominant parasite species, interventions, and outcomes from the included reports using a standardized data collection form. When available, the follow-up length and relapse rate were also recorded. The main outcomes studied were clinical cure, defined as complete ulcer healing, and relapse and subsequent mucosal involvement rates.

The quality of the randomized studies was evaluated using the following criteria: 1) double-blind; 2) concealment of treatment allocation; 3) blinding of outcome assessment; and 4) intention-to-treat analysis. Concealment of treatment allocation was adequate if the patients and enrolling investigators could not predict assignment. Outcome assessment was considered blinded if the investigator who assessed the outcome had no knowledge of the treatment assignment. The analysis was performed according to the intention-to-treat principle, if all randomized patients were included in the analysis and maintained in their originally assigned groups. If there was not sufficient information to assess the quality of the study, it was deemed inadequate.

The timing of cure assessment after treatment varied significantly among studies: relatively early in some studies, while later in others. Considering that the time between the end of treatment and healing assessment can potentially influence cure rate, we established two cure rate occasions by pooling studies according to the time at which cure assessment was originally performed and by implementing the recommendations for the standardization of outcomes in CL trials [10]. After pooling the studies, two cure assessment time points were included in our analysis: a “initial cure”, assessed at approximately three months, and a “definitive cure”, assessed at 9 months after “no treatment” or placebo use. With the aim of gathering as much information as possible, even if the author had defined cure according to clinical assessment performed at a specific moment, if information was available at other moments of interest, they were included in our analysis.

The efficacy of therapy was assessed by clinical criteria in all studies. The definitions of clinical cure varied slightly between studies, with most studies requiring complete epithelialization of all lesions. Only one study defined cure as 80% or more of the ulcer area being re-epithelialized [11]. Some authors carried out parasitological cure assessment in addition to clinical assessment [12–14]; however, when both outcomes (clinical and parasitological) were independently available, the parasitological findings were not considered in the cure outcome analysis (data no shown). In three studies [15–17], cure was assessed solely at the 6-month follow-up. For studies that performed “initial cure” assessment (at approximately 3 months of follow-up) and also presentedthe lesion evolution without treatment at 6–12 months of follow-up, the second assessment was also included in the cure outcome analysis. However, in many studies [11, 13, 14, 18, 19], patients whose lesions did not achieve healing were removed from the protocol and were treated with conventional drugs, making a “definitive cure” assessment unfeasible. In the majority of studies, patients were followed for approximately 6 to 12 months.

To standardize the cure criteria, even if the authors had considered a negative parasitological finding as criteria of cure, in our analysis, cure was considered exclusively according to clinical criteria. Similarly, some studies [12, 17] included no reactivation of any ulcer through 12 months of follow-up as an additional criteria for cure. As adopted in previous studies, to standardize the outcomes among studies, if a clinical assessment was available, cure was defined exclusively by the clinical criteria (re-epithelialization of the lesion at any given time), regardless of the subsequent observation of relapse.

## Quantitative data synthesis

Comprehensive Meta-Analysis® software v.2.2.048 was used to perform the one-group meta-analysis of study arms using placebo or no treatment to estimate the pooled rates of spontaneous cure according to the intention-to-treat principle, irrespective of how the original study investigators analyzed the data. We used the inconsistency (*I*^*2*^*)* statistic to evaluate heterogeneity and Egger's test to assess publication bias. For all cure and relapse rate analyses, a random model was chosen. To evaluate cure rate stratified by *Leishmania* species, we used a mixed effects analysis—a random effects model was used to classify the studies into each subgroup.

## Results

The PubMed search identified 16 relevant articles. The LILACS database search identified two additional papers. Among the three search strategies performed in the PubMed database, the first one, which was the most wide, was the most sensitive and identified all 13 studies ultimately included in this review. The search strategy that included the “placebo” term was not as sensitive as the first search strategy. One possible explanation for this finding is that studies describing the evolution of untreated patients are not properly indexed. Third search strategy was useful to compare ours results to those retrieved by others published reviews on the same topic. The results of the three searches are shown in a flowchart in S1 Fig. After exclusion based on titles and abstracts, 18 potentially relevant papers were selected for full text evaluation. Of them, 2 papers were excluded, because the same patients were described elsewhere [20, 21]; one study was a review [22] and the other included less than 10 patients in the placebo arm [23]. One additional study [24] was excluded, because the patients in the placebo arm were given a potentially active drug (1% lidocaíne),which was not considered a genuine ‘‘vehicle control”. Thus, we included 13 studies [11–19, 25–28] that involved 352 patients presenting with localized CL who were followed after no therapy or placebo use (Table 1). No study included patients infected with HIV or presenting with other immunosuppressive diseases. All included studies were randomized trials, but they compared different treatment regimens and, in some of them, the patients who were untreated were those who refused therapy or were members of a “historical” control group. The methodological characteristics of the studies, namely the diagnostic, treatment arms (MA: meglumine antimoniate, SS: sodium stibugluconate, MBCL: paromomycin 15% + methylbenzethonium chloride, allopurinoll + probenecid, ketoconazole, miltefosine, cryoterapy, pentamidine, itraconazole and localized heat), inclusion, exclusion and cure criteria are shown in Table 1. In nine studies, the control group received a placebo, and in the remaining four studies, the control group received no treatment.

**Table 1:** Main methodological characteristics of the studies.

Year, author	Study arms (number of patients)	Study sample	Inclusion criteria	Exclusion criteria	Follow-up (months)	Leishmania species characterization (% of patients)	Leishmania species	Cure criteria used	Lost to follow-up
**2013, Soto *et al*.**	MA intralesional (30), Cryotherapy (20),Placebo cream (30)	80	parasitological diagnosis (smears or biopsy, culture), ≥12 years of age, one ulcerative lesion ≤30 mm^2^ resulting in a total lesion area of ≤900 mm^2^	previous anti-leishmanial therapy (< 3 months), mucosal lesions, concomitant diseases	6	42/80 (52.5%)	L. braziliensis (36); L. guyanensis (2); L. amazonensis (2); L. lainsoni (2)	100% reepithelialization within 6 months after the end of therapy	3/80 (3.7%)
**2004, Soto *et al*.**	Miltefosine (89); Placebo (44)	133	parasitological diagnosis (smears, culture, biopsy, monoclonal antibody), ≥12 years of age	previous anti-leishmanial therapy (< 4 weeks), mucosal lesions, concomitant diseases, pregnancy, lactation	6	53/133 (39.8%)	L braziliensis (29); L mexicana (17); L panamensis (7)	100% reepithelialization within 6 months after the end of therapy	8/133 (6%)
**2002, Soto *et al*.**	New drug test # (33), Placebo (12)	45	parasitological diagnosis (smears, culture, biopsy, monoclonal antibody), total ulcer lesion size < 2000 mm^2^, lymphadenopathy < 1 cm	severe disease; papular or nodular lesions; mucosal lesions, concomitant diseases	6	5/45 (11.1%)	L. panamensis (5)	100% reepithelialization by the 6-month follow-up	8/45 (17.8%)
**2001, Arana *et al*.**	Paromomycin + MBCL (38), Placebo (38)	76	parasitological diagnosis (smears, culture), age between 10–60 years (smears, culture)	> 4 lesions; lesion > 5 cm in diameter; previousanti-leishmanial therapy; mucosal lesions, concomitant diseases	12	not available	not available	100% reepithelialization by the 13-week follow-up	8/76 (10.5%)
**1997, Neva *et al*.**	Topical paromomycin+ urea (23); Placebo (30)	53	parasitological diagnosis (culture)	NA	5	53/53 (100%)	L. mexicana (18), L. chagasi (35)	100% reepithelialization within 11 weeks after the end of therapy	6/53 (11.3%)
**1997, Velez *et al*.**	Allopurinol (60), Placebo (56); MA (66)	182	parasitological diagnosis (smears, culture), age between 6–60 years; body weight appropriate for height	Previous anti-leishmanial therapy, lesions close to the eyes; mucosal lesions, concomitant diseases, pregnancy	12	not available	not available	100% reepithelialization within 3 months after the end of therapy with no relapse by the 12-month follow-up	25/182 (13.7%)
**1995, Martha *et al*.**	Allopurinol+probenecid (30); SS (30); No therapy (15)	75	parasitological diagnosis (smears, culture, biopsy, monoclonal antibody)	mucosal lesions, concomitant diseases	12	26/75 (34.7%)	L. panamensis (12); L. guyanensis (7); L. braziliensis (4);L. mexicana (1); L. amazonensis (2)	100% reepithelialization by the 70-day follow-up	13/75 (17.3%)
**1993, Soto *et al*.**	MA (23); Pentamidine (27), Itraconazole(20); No treatment (22)	92	parasitological diagnosis (smears, culture, biopsy, monoclonal antibody), age between 18–60 years	mucosal lesions, concomitant diseases, previous anti-leishmanial therapy	12	not available	not available	100% reepithelialization within 1.5 months after the end of therapy	6/92 (6.5%)
**1992, Martinez *et al*.**	MA (33); Allopurinol+MA (35); Allopurinol (25), No treatment (17)	110	parasitological diagnosis (smears, culture, biopsy); lesions confined to the upper portion of the trunk or arms	allergy to antimony or allopurinol; pregnant or nursing, concomitant diseases, more than 20% above or below ideal weight	12	110/110 (100%)	L. panamensis (110)	100% reepithelialization and negative culture within 3 months after the end of therapy	not available
**1992, Navin *et al*.**	SS (40); Ketoconazole (38), Placebo (40)	120	parasitological diagnosis (smears, culture), male gender	mucosal lesions, concomitant diseases, previous anti-leishmanial therapy	13	97/120 (80.8%)	L. mexicana (32); L. braziliensis (63); both L. mexicana and L. braziliensis (2)	100% reepithelialization by the 13-week follow-up	7/120 (5.8%).
**1991, Guderian *et al*.**	SS (30); Allopurinol+probenecid (30); No treatment (15)	75	parasitological diagnosis (smears, culture, biopsy, monoclonal antibody)	mucosal lesions, concomitant diseases, less than 6 months of follow-up	1.5 *	23/75 (30.7%)	L. panamensis(12); L. guyanensis (5); L. braziliensis(3); L. mexicana(3)	>80% reepithelialization by the 1-month follow-up	14/75 (18.7%).
**1990, Navin *et al*.**	MA (22); Localized Heat (22); Placebo (22)	66	parasitological diagnosis (smears, culture), lesions<25 cm^2^	lesions in ear, near the eye, on the finger; presence of unilateral lymphadenopathy; mucosal lesions, concomitant diseases, previous anti-leishmanial therapy	12	53/66 (80.3%)	L. mexicana (13); L. braziliensis (40)	100% reepithelialization by the 13-week follow-up	3/66 (4.54%)
**1990, Saenz *et al*.**	Ketoconazole (22); SS (19); Placebo (11)	52	parasitological diagnosis (smears, culture)	facial or mucosal lesions; concomitant disease	3	38/52 (73.1%)	L. guyanensis (37); L. mexicana (1)	100% reepithelialization by the 1-month follow-up	NA

The patient characteristics are shown in S1 Table. Patient age ranged from 19 to 36 years; 88% of the patients were male, and most of them had less than three lesions localized in arms or legs. CL was diagnosed in all studies if patients had a compatible clinical illness and *Leishmania* was identified on Giemsa-stained smears, culture or biopsy. In a few studies, a positive monoclonal antibody test [11, 15, 16, 25, 28] was also considered to confirm *Leishmania* infection.Although all studies had required laboratory confirmation of the diagnosis of CL, the percentage of isolation of the *Leishmania* species across studies ranged from 11 to 100% of the patients (112 of 352, 32%). In three studies *Leishmania* specie characterization was not performed.

**Fig 1:**
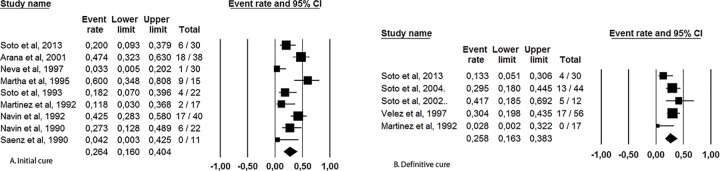
Initial (A) and definite (B) cure rates observed in American cutaneous leishmaniasis patients without treatment or with placebo use.

**Fig 2:**
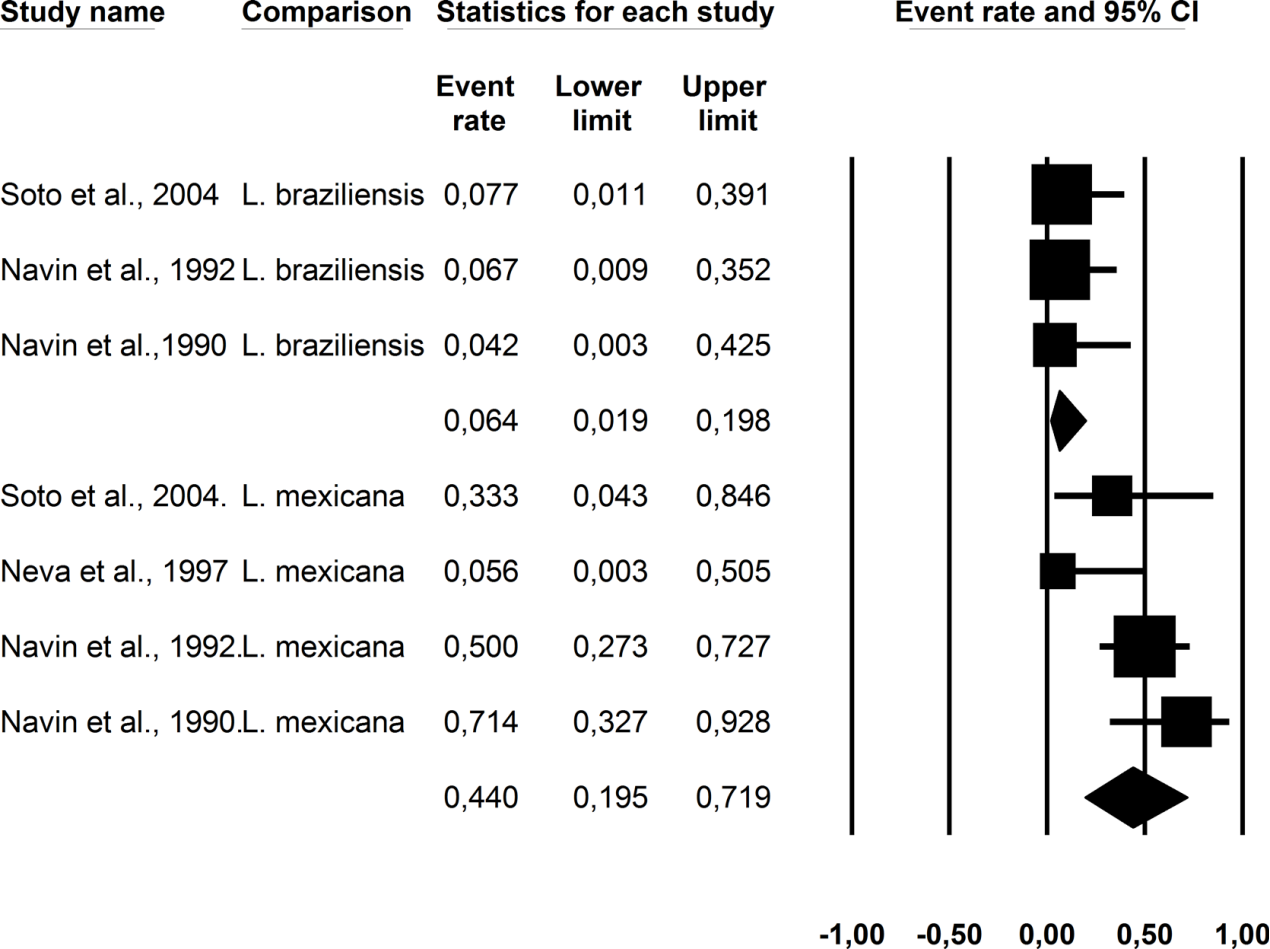
Cure rate observed in American cutaneous leishmaniasis patients without treatment or with placebouse according to *Leishmania* species.

The summarized cure rates according to the intention-to-treat analysis are shown in Table 2 and Figs 1 and 2. The “initial cure” was assessed at a median of 11.5 weeks of follow-up (range: 4.5 to 13 weeks of follow up) and “definitive cure” was assessed at a median of 9 months of follow-up (range: 6 to 12 months of follow-up). Relapse was assessed in patients who were treated and were considered cured. Of note, the study presenting with the highest spontaneous cure rate [11] was the one that used a less stringent criterion for cure (unlike all other studies in which complete re-epithelialization was the requirement, in that study, reepithelization equal to or above 80% of the ulcer area was considered sufficient for cure establishment). Considering the current cure definition of complete reepithelization [10] and the requirement of standardization of criteria for combining the results of multiple studies, we excluded this discrepant study from the final analysis. Using placebo or no treatment, the initialcure ratewas 26% (CI95%: 16 to 40%, heterogeneity I^2^ = 70), and the definitive cure rate was 26% (CI95%:16 to 38%, heterogeneity I^2^ = 48). In addition, despite the smaller number of patients with species characterization available (only 112 of 352), a significantly lower cure rate was observed for *L*. *braziliensis* infection (6.4%, CI95%:0.2 to 20%) than for *L*. *mexicana* infection (44%, CI95%:19 to 72%), p = 0.002.

**Table 2:** Outcome rates observed in American cutaneous leishmaniasis patients without treatment or with placebo use.

Author, year	Patients in placebo or “no treatment” arm (patients)	Initial cure (n/%)	Definitive cure (n/%)	Cure rate according to *Leishmania* species(n/%)	Relapse after cure (n/%)	Late mucosal involvement (n/%)
**Soto *et al*., 2013**	30	6/30 (20)	4/30 (13.3)	Not informed	2/6 (33.3)	Not available
**Soto *et al*., 2004**	44	Not informed	13/44 (29.5)	1/13 (7.7) *L*. *braziliensis* 1/3 (33.3) *L*. *mexicana*	1/13 (7.7)	Not available
**Soto *et al*. 2002**	12	Not informed	5/12 (41.7)	Not available	Not available	Not available
**Arana *et al*., 2001**	38	18/38 (47.4)	Not available	Not available	0/13 (0)	Not available
**Neva *et al*., 1997**	30	1/30 (4.5)	Not available	0/8 (0) *L*. *mexicana* 1/22 (4.5) *L*. *chagasi*	Not available	Not available
**Velez *et al*., 1997**	56	Not available	17/56 (30.4)	Not available	0/17 (0)	1/56 (1.8)
**Martha *et al*., 1995**	15	9/15 (60)	Not available	Not available	0/5 (0)	Not available
**Soto *et al*., 1993**	22	4/22 (18.2)	Not available	Not available	2/4 (50)	Not available
**Martinez *et al*., 1992**	17	2/17 (11.7)	0/17 (0)	0/17 (0) *L*. *panamensis*	2/2 (100)	Not available
**Navin *et al*., 1992**	40	17/40 (4.5)	Not available	1/15 (6.7) *L*. *braziliensis* 8/16 (50) *L*. *mexicana*	3/17 (17.6)	Not available
**Guderian *et al*., 1991**	15	9/15 (60)	Not available	Not available	0/9 (0)	Not available
**Navin *et al*., 1990**	22	6/22 (27.3)	Not available	0/11 (0) *L*. *braziliensis* 5/7 (71) *L*. *mexicana*	0/6 (0)	Not available
**Saenz *et al*., 1990**	11	0/11 (0)	Not available	Not available	Not available	Not available

**Fig 3:**
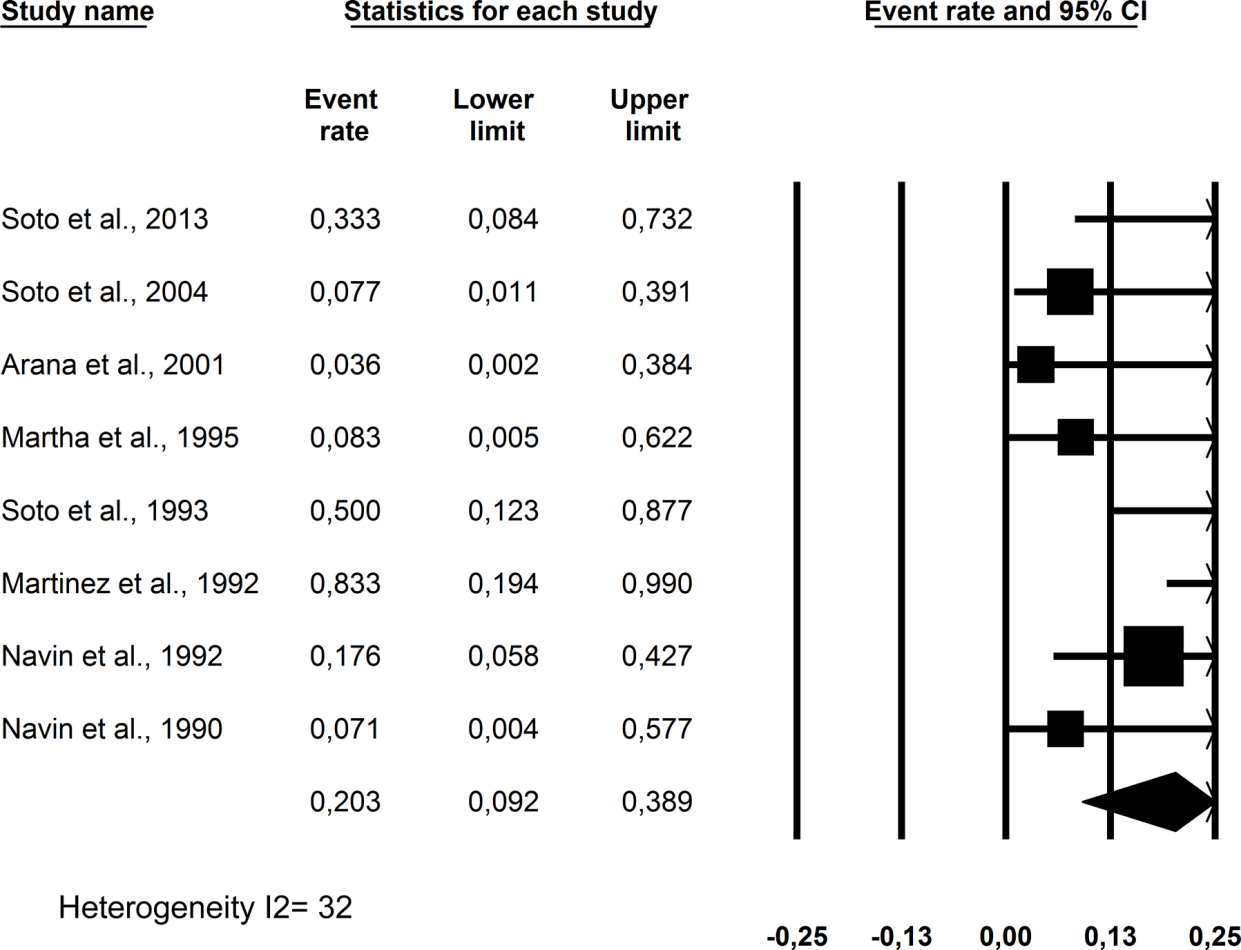
Relapse rate observed in American cutaneous leishmaniasis patients without treatment or with placebo use who presented with initial ulcer healing.

The relapse rate was 20% (CI95%:9.2 to 38.9%) and is shown in Fig 3. The relapse rate is the percentage of patients with worsening skin lesions after initial healing and was usually assessed at approximately 12 months of follow-up.

The study quality assessment is shown in Table 3. In general, the studies included adequate numbers of patients and CL was invariably parasitologically confirmed. These patients were largely representative of the source populations. However, in two studies [11,12], in the placebo or no therapy arm, patients were not randomly allocated, which generates an important selection bias.

**Table 3:** Study quality evaluation.

Author, year	Double-blind	Concealment of treatment allocation	Blinding of outcome assessment	Intention-to-treat analysis	Comments
**Saenz *et al*., 1990**	No	No *	No	Yes	* Randomization generation domain not informed. The placebo group was sequentially recruited one year after the recruitment of the treatment groups.Three patients were not randomized and received ketoconazole.
**Navin *et al*., 1990**	No	No*	No	Yes	*Randomization generation domain not informed
**Guderian *et al*., 1991**	No	No*	No	No	*Randomization generation domain not informed
**Martinez *et al*., 1992**	No	No*	No	Yes	* Randomization generated by a computer program. Patients who agreed to participate but refused to receive injections received allopurinol. Patients not eligible for treatment were allocated to the control group. These groups were not randomized but were self-selected.
**Navin *et al*., 1992**	No	Yes*	Yes	No	*Randomization generated by a computer program
**Martha *et al*., 1995**	No	No*	No	No	*Randomization generation domain not informed
**Soto *et al*., 1993**	No	No*	No	No	*Numerical sequence randomization. Randomization was not uniform for all patients included. Twenty of 92 patients were randomized to one of the treatment arms, without the placebo option.
**Neva *et al*., 1997**	Yes	Yes	Yes	Yes	Randomization generated by a computer program
**Velez *et al*., 1997**	Yes*	No**	Yes***	Yes	*Only the allopurinol and placebo groups were double-blind, not the *Glucantime* group. **Randomization generation domain not informed. ***Yes to placebo and allopurinol groups, not mentioned for *Glucantime* group.
**Arana *et al*., 2001**	Yes	Yes	Yes	No	Tubes randomly numbered by industry
**Soto *et al*., 2002**	No	No*	Yes	Yes	*Randomization generation domain not informed.
**Soto *et al*., 2004**	Yes	No*	Yes	Yes	* Randomization generation domain not informed.
**Soto *et al*., 2013**	No	Yes*	No**	Yes	* Deck of cards randomization. **Information not available

## Discussion

Our main result was the very low spontaneous (6%) healing rate observed in American CL caused by *L*. *braziliensis*. This CL cure rate significantly contrasts with that described with antimonial derivatives in the Americas (71.3–91.4%) [6]. Considering all patients gathered in this study, the spontaneous cure rate was also low (26%); however, there was high heterogeneity across studies (IC95%: 16–40%), preventing us from expanding this conclusion to all species that cause CL in the Americas.

Although we have made an extensive review by combining several search strategies, our analysis includes few studies and a relatively small number of patients thus hampering a stratified analysis of cure by *Leishmania*s pecies. Another limitation is the bias represented by the imperfect randomization of patients. Despite these limitations, this review raises necessary reflection about ethical aspects in CL trials.

The three most common reasons used to justify the use of a placebo arm in American CL studies are as follows: 1) the doubtful cure rate obtained with pentavalent antimonial drugs, as the current evidence of their efficacy is not sufficiently strong, mainly because of the weakness of clinical trials; 2) the non-systematic observation of spontaneous healing in CL, which can “contribute” to the cure rate outcome of a investigated treatment, resulting on an over-estimated cure rate and 3) the benign nature of the damage caused by delayed treatment or no treatment or placebo, frequently considered to be reversible and “not serious” harm.

Considering the previously published systematic reviews about the efficacy of the antimony derivatives and the results presented here, the first two justifications for the use of a placebo arm in American CL studies can be considered, in the present state of knowledge, to be negated as follows: the available therapies normally prescribed have recognized efficacy, and the spontaneous cure rate for CL is low in the Americas. Therefore, what still needs to be examined is the magnitude of harm caused by the disease or how much suffering patients can ethically be exposed to by delaying appropriate treatment with placebo administration.

There are many reasons for the historical use of placebo, including the endorsement provided by regulatory bodies in the interests of accuracy [29]. The most efficient way to eliminate the effect of placebo in the final statistical analysis is random allocation of patients into a placebo concurrent control arm. Although this approach is scientifically sound, ethical concerns arise under specific conditions that outweigh the alleged benefits of this protocol design.

There is agreement that the use of placebo or another form of untreated control is almost always unethical when a therapy is available that has been shown to improve survival or decrease serious morbidity. However, for conditions in which the treatment does not affect the patient’s long-term survival, as is the case for CL, this discussion becomes enormously complex and causes division among researchers. It is well noted that the Helsinki Declaration accepts placebo-controlled trials since its 2008 revision; a decision that was maintained in the 2013 version [30]. This World Medical Association position is far from being generally accepted, and the Brazilian National Health Board is against these two revisions and keeps the position that placebo-controlled trials are unethical whenever they are used to evaluate new treatments for conditions when proven effective treatments exist [31]. In countries where the Helsinki Declaration is adopted, researchers and research organizations should observe the two conditions that it states as necessary for the use of a placebo or no intervention arm: the existence of “compelling and scientifically sound methodological reasons” and “the patients who receive any intervention less effective than the best proven one, placebo, or no intervention will not be subject to additional risks of serious or irreversible harm”. According to Emanuel and Miller (2001) [32], a placebo-controlled trial has a sound scientific rationale if the following criteria are met: “there is a high placebo-response rate; the condition is typically characterized by a waxing-and-waning course, frequent spontaneous remissions, or both; and existing therapies are only partly effective or have very serious side effects; or the low frequency of the condition means that an equivalence trial would have to be so large that it would reasonably prevent adequate enrollment and completion of the study”. In a simple analysis, it is noted that the above criteria cannot be fully applied to CL and the “compelling methodological reasons” that could justify this choice are at least questionable. Furthermore, even when these methodological criteria are met, the risk of using a placebo control should also be evaluated according to several ethical criteria. The Helsinki Declaration states that research participants in the placebo group “should not be substantially more likely than those in the active-treatment group to die; to have irreversible morbidity or disability or to suffer other harm; to suffer reversible but serious harm; or to experience severe discomfort” [33]. Based on that statement, Temple and Ellenberg [34] claimed that the use of a placebo control is ethical if the research participants who receive the placebo will experience “no permanent adverse consequence”, if there is only a risk of “temporary discomforts,” or if they “will not be harmed.” Ultimately, it is notable that “scientific rationale criteria” for placebo use permit suffering of the study participants that is not negligible, and the interpretation of these “no equivalent principles” is a very arduous task. In addition, parameters, such as the seriousness of the damage and the degree of discomfort, are too subjective and personal to allow for arbitration.

It is also useful to review the risks addressed by the U.S. Federal regulations [32],which can be used to categorize the risks of damage to the subjects into one of three types: physical, psychological, and informational. Furthermore, the proponents of placebo controls focus exclusively on physical harm. It is important to note that skin lesions are a common cause of social isolation, disruption of relationships and loss of employment. These social harms lead to emotional damage, both of which are either not considered or are dismissed. However, as noted by Emanuel and Miller [32],in a contradictory manner, “psychological and social harms are invoked to justify the value of the research”.

Another important topic linked to the placebo discussion is the toxicity related to antimonial derivatives, an argument that is sometimes used to promote placebo use. While we emphasize that the toxicity of antimony derivatives is not negligible and that, in CL, the “best proven treatment” is a “non-optimal treatment”, we also recognize that unexpected adverse effects are very rare in young patients without comorbidities, who normally compose the study groups of CL studies, and that monitored antimony use can avoid much of the serious adverse events reported [35]. It is also important to note that antimony derivatives are considered to have high evidence quality in the treatment of CL and are strongly recommended in the guide produced by the Pan American Health Organization for the treatment of leishmaniasis [36]. Obviously, there are other operational implications of using antimony derivatives: the use of an antimony derivative under close adherence to the recommended precautions as comparator arm in clinical CL trials potentially restricts recruitment, and the study may require increased time and funds.

Finally, in American CL, this discussion brings up another important and unclear point: mucosal involvement is a late complication that is mainly related to *L*. *braziliensis* infection but also to species found in the Americas, including *L*. (*V*.) *panamensis*, *L*. (*V*.) *guyanensis and L (L) amazonensis* [37]. Unfortunately the studies available and included in this review did not perform a sufficiently long follow-up preventing us to conclude about the mucosal involvement risk. A detailed analysis of this issue was not the purpose of the present discussion; however, despite reports of this complicationeven after appropriate therapy, the risk seems to increase, moreso thanthe risks of any other complications, in patients who are not adequately treated [38, 39].

Considering these unknown elements and the risks embedded in each study design, research planning, particularly in the CL field, requires deep ethical reflection. It is certainly necessary to review the recommendations for clinical trials focused on American CL, especially when the infectious agent is *L*. *braziliensis*. Based on a low spontaneous cure rate observed in non-treated patients and our above reflections, we believe that for clinical studies of CL in the Americas, there is a sufficient basis for discouraging the use of a placebo or non-treatment arm. Patients need to be protected from unethical exposures to morbidity, pain and social stigmatization and, instead, should be offered immediate and adequate treatment.

## Supporting Information

S1 Fig Review search strategy. (TIF)

S1 Table Population characteristics of the placebo or no therapy arm. (DOCX)

S1 Text PRISMA checklist. (DOC)
