# Supplementary material for: The Cure Rate after Placebo or No Therapy in American Cutaneous Leishmaniasis: A Systematic Review and Meta-Analysis
Source: PLoS One. 2016 Feb 19;11(2):e0149697. doi: 10.1371/journal.pone.0149697 (PMC4760744; doi:10.1371/journal.pone.0149697)
Supplement: S1 Table — (DOCX) [file pone.0149697.s002.docx]

Population characteristics of the placebo or no therapy arm

| Year, author | Age (mean, years) | Gender male/female | Lesion number (mean) | Lesion site (number of patients) | Lesion size  (mm^2^) | Lesion duration (months before therapy) |
| --- | --- | --- | --- | --- | --- | --- |
| **2013, Soto *et al.*** | 32±13 | not available | not available | members (24); head (6) | 188±145 | not available |
| **2004, Soto *et al.*** | 26.5± 12.5 | 38/ 6 | 1 (median) | not available | not available | not available |
| **2002, Soto *et al.*** | 26 ± 9 | 45/0 | 1.6 (standard deviation not available) | not available | 203 ± 259 | not available |
| **2001, Arana *et al.*** | 20.3±0.8 | not available | 1.3 ±0.1 | members (32); trunk (3); other (3) | 130±0.3 | not available |
| **1997, Neva *et al.*** | not available | not available | not available | not available | not available | not available |
| **1997, Velez *et al.*** | 25±13 | 35/21 | 3.3±3.4 | upper body (25); lower body (16); upper and low members (15) | not available | 2.7±1.9 |
| **1995, Martha *et al.*** | 34 (standard deviation not available) | 8/7 | 1.4±0.73 | head (2); trunk (2); members (11) | 130 * | not available |
| **1993, Soto *et al.*** | not available | 22/0 | 1.9 ± 1.47 | not available | 331 ± 66 | 2.5±0.4 |
| **1992, Martinez *et al.*** | 21 (standard deviation not available) | 17/0 | 1.76 ±0.75 | head (2); arms (14); trunk (1) | not available | not available |
| **1992, Navin *et al.*** | 21.3±1.4 | 40/0 | 1.5±0.2 | not available | 200±40 | 1.97±0.23 |
| **1991, Guderian *et al.*** | 36 (standard deviation not available) | not available | 1.3 (standard deviation not available) | not available | 113±12 | 3.2 * |
| **1990, Navin *et al.*** | 19.4±0.5 | 22/0 | 1.5±0.2 | not available | 490±140 | 2.1±0.33 |
| **1990, Saenz *et al.*** | 31 (standard deviation not available) | 11/0 | 2.1±1.2 | members (22); head (1); trunk ( 0) | 95 ±77 | 1.85±0.62 |
